# Music and Language Syntax Interact in Broca’s Area: An fMRI Study

**DOI:** 10.1371/journal.pone.0141069

**Published:** 2015-11-04

**Authors:** Richard Kunert, Roel M. Willems, Daniel Casasanto, Aniruddh D. Patel, Peter Hagoort

**Affiliations:** 1 Max Planck Institute for Psycholinguistics, Nijmegen, The Netherlands; 2 Radboud University Nijmegen, Donders Institute for Brain, Cognition and Behavior, Nijmegen, The Netherlands; 3 Psychology Department, University of Chicago, Chicago, Illinois, United States of America; 4 Tufts University, Medford, Massachusetts, United States of America; Massachusetts Institute of Technology, UNITED STATES

## Abstract

Instrumental music and language are both syntactic systems, employing complex, hierarchically-structured sequences built using implicit structural norms. This organization allows listeners to understand the role of individual words or tones in the context of an unfolding sentence or melody. Previous studies suggest that the brain mechanisms of syntactic processing may be partly shared between music and language. However, functional neuroimaging evidence for anatomical overlap of brain activity involved in linguistic and musical syntactic processing has been lacking. In the present study we used functional magnetic resonance imaging (fMRI) in conjunction with an interference paradigm based on sung sentences. We show that the processing demands of musical syntax (harmony) and language syntax interact in Broca’s area in the left inferior frontal gyrus (without leading to music and language main effects). A language main effect in Broca’s area only emerged in the complex music harmony condition, suggesting that (with our stimuli and tasks) a language effect only becomes visible under conditions of increased demands on shared neural resources. In contrast to previous studies, our design allows us to rule out that the observed neural interaction is due to: (1) general attention mechanisms, as a psychoacoustic auditory anomaly behaved unlike the harmonic manipulation, (2) error processing, as the language and the music stimuli contained no structural errors. The current results thus suggest that two different cognitive domains—music and language—might draw on the same high level syntactic integration resources in Broca’s area.

## Introduction

Music and language are uniquely human abilities which, despite their obvious differences, appear to share more than just a common population of users. Specifically, it has been proposed that one overlapping aspect is found in syntactic processing [1]. Syntactic processing—whether in language or in music—involves the integration of discrete elements (e.g., words, tones/chords) into higher order structures (e.g., sentences in language and harmonic sequences in music) according to a set of combinatorial principles that are implicitly understood by members of a culture [1]. Using functional magnetic resonance imaging (fMRI), the present study aimed to find neural evidence for shared syntactic integration resources recruited by both music and language.

In the present study we defined music syntax processing as harmonic structure processing, in line with many previous studies (e.g., [2,3]). Harmony in Western tonal music refers to the organization of pitches in terms of scales, chords, and keys. The basic ‘pitch material’ of Western tonal/harmonic music (henceforth, tonal music) consists of 12 pitches per octave, each representing one of 12 octave-equivalent ‘pitch classes’ (e.g., all the C-notes on a piano keyboard). When playing in a musical ‘key’, a subset of 7 out of 12 pitch classes (in-key tones) is emphasized. Therefore, once a listener has derived a sense of key, e.g., C-major, from a musical piece (for a computational model see [4]) she or he expects certain tones—for example in-key tones such as C—more strongly than others—out-of-key tones such as C# [5,6]. Thus, in tonal music, incoming tones are evaluated in terms of a harmonic framework into which they are continuously integrated.

Do musical and linguistic syntactic processing overlap in the brain? On the one hand, it is known that sensitivity to linguistic syntax and to tonal harmony can dissociate after brain damage, suggesting independence of these two domains (e.g., [7]). On the other hand, there is evidence that linguistic syntactic processing and tonal harmonic processing involve similar brain responses [2,8–10](for a review see [11]). To resolve this paradox, the ‘Shared syntactic integration resource hypothesis’ or SSIRH [1] posited a distinction between domain-specific representations in long-term memory (e.g., stored knowledge of words and their syntactic features, and of chords and their harmonic features) and shared neural resources which act upon these representations as part of structural processing. This “dual-system” model considers syntactic processing to involve the interaction (via long-distance neural connections) of “resource networks” (hypothesized in frontal brain regions) and “representation networks” (hypothesized in temporal brain regions). Patel [1] posited that resource networks are recruited when structural integration of incoming elements in a sequence is costly; that is, when it involves the rapid and selective activation of low-activation items in representation networks. Cognitive theories of syntactic processing in language (dependency locality theory; [12]) and of tonal harmonic processing in music (tonal pitch space theory; [13]) were used to specify the notion of processing cost. In both models, incoming elements incur large processing (activation) costs when they need to be mentally connected to existing elements from which they are “distant” in a cognitive sense (e.g., in music, distant in tonal pitch space rather than in terms of physical distance in Hz; in language, distant in terms of the number of intervening words between a syntactic head and the to-be-integrated word). According to the SSIRH, in such circumstances, activity in frontal brain regions increases in order to rapidly activate specific low-activation representations in temporal regions via reentrant connections. Put another way, music and language share limited neural resources in frontal brain regions for the activation of stored structural information in temporal brain regions (for a similar model specific to language see [14,15]).

The SSIRH predicts that since neural resources for structural integration are limited, simultaneous costly integrations in harmony and language should lead to interference. Testing this prediction requires experiments which present music and language simultaneously, and which align points of difficult structural integration in the two domains. This prediction has been supported in several studies which presented chord sequences and sentences (two using ERPs [16,17] and two using behavioral methods [18,19]) or melodies and sentences (one using ERPs [20] and one using behavioral methods [3]), see [21] for an overview. For example, the behavioral study of Fedorenko et al. [3] (which informed the design of the current neural study) manipulated linguistic syntactic integration difficulty via the distance between dependent words. These researchers manipulated the structure of embedded relative clauses as shown below (italicized):

(a) The boy *that helped the girl* got an “A” on the test.
(b) The boy *that the girl helped* got an “A” on the test.

The sentences were sung to melodies (one note per word) which did or did not contain an out-of-key note on the last word of the relative clause: ‘girl’ in (a), ‘helped’ in (b). According to dependency locality theory [12], this word is associated with a distant structural integration in (b) (between ‘helped’ and ‘that’) but not in (a). A control condition was included for an attention-getting but non-harmonically deviant musical event: a 10 dB increase in volume on the last word of the relative clause. After each sentence, participants were asked a comprehension question, and accuracy was assumed to reflect processing difficulty. The results revealed an interaction between musical and linguistic processing: comprehension accuracy was lower for sentences with distant versus local syntactic integrations (as expected), but crucially, this difference was larger when melodies contained an out-of-key note. The control condition (loud note) did not produce this effect: the difference between the two sentence types was of the same size as that in the conditions which did not contain an out-of-key note.

However, the brain areas underlying such interaction effects are unclear. Overall, a great number of brain lesion, electrophysiological and hemodynamic brain imaging studies converge in highlighting one key region for syntax processing in either music or language when studied separately: Broca’s area [9,22–26]. Thus this region may be the locus of the interaction effect, either in the left hemisphere and/or in the right hemisphere homologue of this area [9,22,24,27,28].

In searching for interactions between language and music in Broca’s area, the current study was mindful of a confound identified by Rogalsky et al. [29]. Many previous experiments using brain measures have operationalized syntactically challenging processing in language as syntactic violation processing [16,17,20,30]. Therefore, general error processing may be shared between music and language, rather than syntactic processing. We used a language manipulation and a music manipulation which did not involve syntactic violations.

Motivated by the hypothesis that Broca’s area was a neural site of interaction between linguistic and musical syntactic processing, the present study specifically focused on the activation pattern of Broca’s area and its right hemisphere homologue in response to structural manipulations of music and language. Participants heard songs containing either a syntactically easy construction containing only a local dependency (SR: subject-extracted relative clause) or a difficult construction containing a non-local dependency (OR: object-extracted relative clause; see [31]). Sentences were sung *a cappella* and the critical word which disambiguated between these two linguistic options was either sung on a regular tone (in-key tone which is easy to integrate in the prevailing harmonic context) or on an irregular tone (out-of-key tone which is not easy to integrate harmonically). Thus, the time point of integration difficulty in music was aligned with the one in language.

Note that neither integration difficulty involved errors. Both types of sentences used in the current study were fully grammatical, and differed in syntactic complexity. Similarly, the use of an out-of-key tone in some of the musical melodies increased their complexity in terms of tonal-harmonic structure [32], but such tones would not be considered ‘errors’ because they are common stylistic elements in tonal melodies. For example, the melodies of Schubert’s lieder often contain out-of-key notes, which are considered to play an important role in the pattern of tension and resolution within the melodies [33].

As noted above, a previous behavioral study in English using a similar design showed an interaction between linguistic and musical conditions in terms of sentence comprehension [3]. As in that study, we included a control condition involving a non-syntactic auditory anomaly—presenting the critical tone in-key but 10dB SPL louder—in order to rule out the possibility that any acoustic irregularity would elicit the predicted interaction. (This loudness increment was identical to that used in [3].)

It was hypothesized that Broca’s area would be sensitive to the increased processing difficulty of a concurrent syntactic integration challenge in both music and language. Furthermore, this brain area is not predicted to be sensitive to the interaction between language syntax and a perceptually salient loudness increase at the critical sentence position, as the latter is not syntactic in nature but instead merely acoustic.

## Materials and Methods

### Ethics Statement

Written informed consent was obtained from all participants prior to measurement and the study received ethical approval from the local reviewing committee ‘‘CMO Arnhem Nijmegen” (CMO no 2001/095 and amendment ‘‘Imaging Human Cognition” 2006, 2008), in accordance with the Research involving human subjects Act, following the principles of the Declaration of Helsinki.

### Participants

19 healthy participants were included in the final analysis (mean age = 22 years, range 18–27). No subject had a known history of neurological, language related or hearing problems and all had normal or corrected-to-normal vision. Five additional participants were excluded due to technical difficulties or excessive movement. The remaining 7 men and 12 women were all right handed, native speakers of Dutch with little formal musical training (mean training = 1.9 years, *SD* = 2.3). All were naïve as to the purpose of the study and were paid for their participation.

### Stimuli

The stimuli were constructed in a fully factorial design. The language dimension had two levels: either a stimulus sentence included a subject-extracted relative clause (SR) or an object-extracted relative clause (OR), as shown in (1). The music dimension had three levels: a melody included either only in-key tones (in-key), or only in-key tones except for one tone which was out-of-key (out-of-key), or only in-key tones with one tone being sung unusually loudly (auditory anomaly). This resulted in 120 stimulus sextuplets: 120 sentences in two linguistic versions and three musical versions, totaling 720 stimuli (120 × 2 × 3). Example stimuli can be accessed online: https://sites.google.com/site/rikunert/CV/example_stimuli_kunert_willems_casasanto_patel_hagoort.

(1)

(1a) Subject-extracted (SR)
  De atleet die de minnaressen opmerkte keek uit het raam.

*Literal*: *The athlete*_*singular*_ *that the mistresses*_*plural*_ *noticed*_*singular*_ *looked*_*singular*_ *out of the window*.

*English translation*: *The athlete that noticed the mistresses looked out of the window*.

(1b) Object-extracted (OR)
  De atleten die de minnares opmerkte keken uit het raam.

*Literal*: *The athletes*_*plural*_ *that the mistress*_*singular*_ *noticed*_*singular*_ *looked*_*plural*_ *out of the window*.

*English translation*: *The athletes that the mistress noticed looked out of the window*.

The language materials consisted of 120 Dutch sentences each in two versions, as can be seen in (1): the critical relative clause verb (‘opmerkte’) agreed in number either with the matrix clause noun phrase (‘De atleet’) in the subject-extracted version or with the relative clause noun phrase (‘de minnares’) in the object-extracted version. By ensuring that these two noun phrases differed in grammatical number we forced the listener to disambiguate the sentence and interpret it as one of the two syntactic versions. Disambiguation was only possible at the moment of listening to the relative clause verb.

Sentences were on average 10 (standard deviation = 1.3) words long with the disambiguating relative clause verb always being the sixth word. The final syllable of the relative clause, which distinguishes between the SR and OR versions, was sung on any beat within a 4/4 bar (11.6% on the first beat, 31.7% on the second, 24.2% on the third, 6.7% on the final beat, the remainder on off-beat notes). The matrix subject was plural in half of the SR sentences, i.e. the grammatical number of the first noun phrase was not indicative of the linguistic condition.

In order to ensure that participants would process the full sentences, a linguistic task checked language comprehension by use of prompts relating to some part of the stimulus sentence (e.g., ‘Iemand merkte de atleet op.’ *Somebody noticed the athlete*.). Prompts required a true/false response. Half the comprehension prompts checked for matrix clause understanding. The other half focused on the relative clause (as in the aforementioned example). In order to avoid task-specific strategies we also created (1) more challenging passive voice prompts and (2) prompts with ‘someone’ (‘iemand’) as a singular subject possibly representing either a plural or a singular noun phrase in the song (see example prompt). Within each comprehension prompt version half the prompts matched the content of the songs.

**Fig 1:**
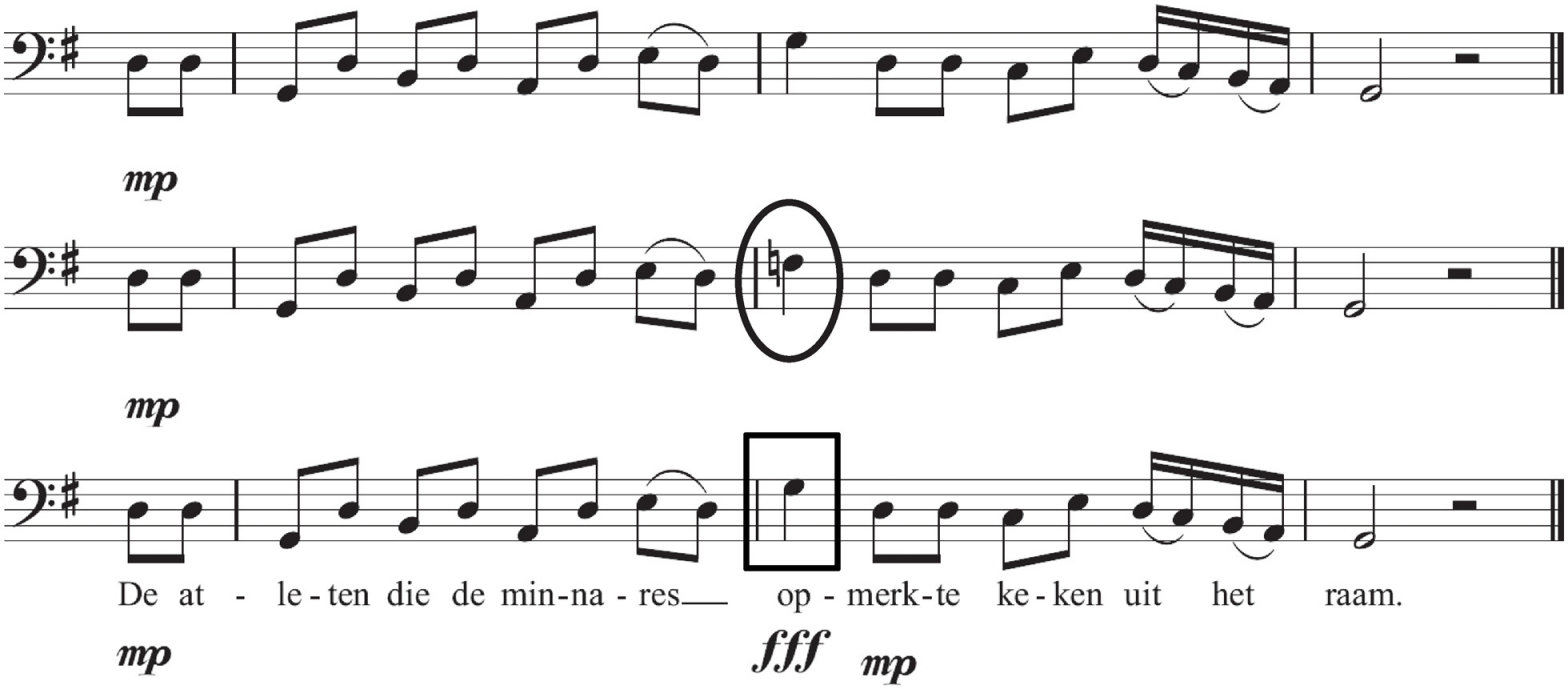
Example stimuli. The top melody shows the in-key condition in which no note is out-of-key (all notes are in G-major). The middle melody shows the out-of-key condition in which only the tone coinciding with the stressed syllable of the relative clause verb (circled) is out-of-key. The bottom melody shows the auditory anomaly condition in which all notes are in G-major but the critical tone is 10dB louder (boxed). The lowest pitch used across all melodies was F#2 (92.5 Hz) and the highest was E4 (329.6 Hz). The Dutch sentence in the figure means: *The athletes that the mistress noticed looked out of the window*.

Each of the two sentence stimulus versions was combined with three versions of a melody (in-key, out-of-key, auditory anomaly). All melodies were composed specifically for this study by a professional composer (Jason Rosenberg, www.jasonrosenberg.org). The three music versions of each of the 120 melodies differed only in terms of the tone sung on the stressed syllable of the disambiguating relative clause verb in terms of pitch (in-key versus out-of-key) or loudness (in-key normal volume versus in-key auditory anomaly [loud volume]), see Fig 1. The in-key and auditory anomaly conditions did not differ in pitch. Melodies were rhythmically diverse and on average 10.2 seconds long (standard deviation = 1.3) at a tempo of 70 beats per minute, i.e. a quarter note corresponded to a nominal duration of 857 ms. The beginning of each melody established a strong sense of key. The three music conditions were in the same key and differed only by one note. This critical tone coincided with the stressed syllable of the relative clause verb, and was either part of the established key (in-key normal volume, auditory anomaly [in-key but loud volume]) or not (out-of-key normal volume). The melodies were composed in such a way that the location of the out-of-key note was musically plausible from the standpoint of harmonic tension-resolution patterns [33]. Rhythmically, the critical note was always a quarter note in length, and occurred on various beats (44.2% on the first beat, 36.7% on the second, 6.7% on the third, 12.5% on the fourth beat). Each of the twelve major keys was used 10 times (10 × 12 = 120 sets). Melodies were in the baritone range.

After stimulus design, the 120 (sets) × 2 (SR and OR versions) × 2 (in-key and out-of-key versions) sung sentences were recorded in a soundproof room at the Max Planck Institute for Psycholinguistics in Nijmegen by a 34 year old male Dutch baritone. The singer was an amateur (Jan-Mathijs Schoffelen) who had been trained for 16 years in total (piano and voice). First, each of the 480 songs (four per set) was recorded separately in each of the linguistic and harmonic conditions. Afterwards, all recordings were normalized for loudness level. Next, steps were taken to control for acoustic cues prior to the critical verb. Specifically, we cut out the verb recording of one harmonic version and pasted it into the audio stream of the other. This created two harmonic versions of each sentence with identical recordings except for the critical verb. After this splicing step the new song signal was adjusted in order to avoid the audibility of the verb recording exchange. To exclude any possible systematic influence of this processing step it was ensured that an equal number (exactly half) of in-key and out-of-key recordings were left unchanged. Next, the auditory anomaly condition of each sentence was created. Of the resulting four files the in-key versions were chosen and the critical tone’s loudness was increased by 10 dB following [3]. All audio manipulations were done with the program Audacity version 1.3 (audacity.sourceforge.net).

### Procedure

Of each of the 120 stimulus sextuplets, each participant heard both linguistic versions, i.e. a total of 240 trials (120 × 2). However, each linguistic version of a stimulus sextuplet was only presented in one music condition. Still, overall, each participant heard an equal number of trials in each music condition. Following an event-related design, the stimuli were ordered pseudo-randomly with the following constraints: (1) no more than three consecutive trials with the same prompt condition (the prompt matches the sentence or not), (2) no more than three consecutive trials of the same music-language condition, and (3) at least ten trials between any stimulus set’s SR and OR versions. For every three participants a new pseudo-randomized stimulus order was used. Within each such participant-triplet, for each trial the musical condition was counterbalanced. The stimuli were presented to the participants using MR-compatible non-magnetic earphones (Sensimetrics, model S14) which also dampened scanner noise. Volume was set at a subject-specific, comfortable level before the start of the experiment.

Participants were asked to concentrate on the linguistic dimension of the sung sentences. As in most previous studies examining interactions between linguistic and musical syntactic processing (e.g., [16,18]), there was no musical task. That is, we relied on the musical structure being processed implicitly. The experiment was organized as follows. Four example trials preceded the experimental session. Experimental trials were divided into eight blocks of 30 sung sentences. After four blocks participants could rest for approximately ten minutes while an anatomical MRI scan was acquired.

Each trial was organized as follows. After a stimulus was played a comprehension prompt was displayed visually through a projector from outside the scanner room. Subjects saw it through a nonmagnetic mirror attached to the head-coil. Within 10 seconds they had to press a button to indicate whether the prompt was true according to the preceding sung sentence or not. Except for the example trials, no feedback was given. Stimulus onset was jittered with respect to volume acquisition by randomly varying the intertrial interval (time between response to the previous trial’s prompt and the song-onset of the next trial) between 3.5 and 6 seconds. During the intertrial interval as well as during the song presentation a fixation cross was displayed centrally. An experimental session lasted approximately 100 minutes.

### fMRI Data Acquisition

The experiment was carried out in a 1.5 Tesla MRI scanner (Siemens Avanto, Siemens Medical Systems, Erlangen, Germany). Thirty-three axial slices were acquired (3.5 mm × 3.5 mm in-plane resolution, 3 mm slice thickness, 0.51 mm slice spacing, field of view [FOV] = 224 mm) covering the whole brain. We used a single-shot echo-planar imaging (EPI) sequence (repetition time [TR] = 2140 ms, echo time [TE] = 40 ms, 90° flip-angle [FA]). In the middle of the scanning session a 3-D T1 scan was acquired (176 slices, voxel size = 1 mm × 1 mm × 1 mm, TR = 2250 ms, TE = 2.95 ms, FA = 15°, sagittal orientation).

### fMRI Data Analysis

Analysis was carried out using SPM8 (www.fil.ion.ucl.ac.uk/spm). The first five volumes of each functional run were discarded. In order to compensate for small head movements, images were realigned to the first image by means of rigid body registration. Slice timing correction was applied by means of linear interpolation to the onset of the first slice. All functional datasets were individually co-registered using the participants’ individual high-resolution anatomical images. Afterwards, this co-registered EPI dataset was normalized to Montreal Neurological Institute (MNI) space. The time series were high pass filtered with a cut-off frequency of 128 seconds and images were spatially smoothed using an 8mm FWHM Gaussian kernel.

The statistical evaluation was performed using the general linear model. The model was generated with a synthetic hemodynamic response function modeled on the manipulated song region, i.e. the start of the critical verb until the end of the song. We separately modeled the six conditions of interest and included two nuisance regressors (dummy variables for run1 and run2) to capture the effect of functional scanning run as well as 18 nuisance regressors derived from the motion correction algorithm. These modeled variability in all three rotations and all three translations due to linear motion, quadratic motion and the first derivative of linear motion (6 motion types × 3 quantifications = 18 regressors; see [34]). Statistical analysis was performed by computing contrast maps for each condition for each participant separately including all of his or her trials (independent of behavioral performance), and the subsequent group analysis involved calculating interaction and main effects in a full factorial ANOVA with factors language (SR, OR) and music (in-key, out-of-key, auditory anomaly). In this way participant was treated as a random factor (‘random effect analysis’). The multiple comparisons problem ensuing from this massive univariate approach was dealt with by applying a topological feature based false discovery rate correction at the .05 level (peak-based FDR) [35,36].

The region definitions used in the structural region of interest (ROI) analysis we derived from the Automated Anatomical Labeling library [37]. The chosen ROIs were those where overlapping activation sites between music harmony and language syntax had been reported (see Introduction): bilateral inferior frontal gyrus (IFG) pars opercularis and pars triangularis, i.e. Broca’s area and its right hemisphere homologue. The Marsbar ROI toolbox version 0.42 [38] was used to derive average contrast values across the 3567 and 3550 voxels of size 2 × 2 × 2 mm^3^, in the left and right structural ROIs respectively, based on data generated during the first level analysis with SPM8. Please note that we are aware of the literature describing structural and functional differences between different parts of Broca’s area (e.g., [23]). Nonetheless, we only defined a single Broca’s area ROI for three reasons: 1) Patel’s SSIRH does not specify which part of Broca’s area should show the predicted interaction between music and language, 2) previous studies which investigated music and language separately found syntax-processing related activations in both pars opercularis (music: [24]; language: [59]) and pars triangularis (music: [9,24]; language: [60]), 3) we aimed to reduce the number of ROIs in order to have sufficient statistical power after controlling for the number of comparisons (Bonferroni method), i.e. the number of structural ROIs.

The ROI data were not normally distributed. Using the SPSS implementation of the Kolmogorov-Smirnov test to check the distributions of OR-SR difference scores within each music condition and ROI revealed that two distributions were significantly different from normal [left and right hemisphere, out-of-key: *D*s_(19)_ > .19, *p*s < .05]. In order to maximize power, these *p*-values of the normality test are not corrected for multiple comparisons. In order to account for the non-normal data distribution, inferential analyses of the ROI data were carried out using random permutation based tests which require no parametric assumptions. In terms of the dependent *t*-tests this amounts to creating a null hypothesis *t*-distribution by randomly applying condition labels to data points within each participant 50,000 times and testing the effect of interest on the randomized data each time. The proportion of randomly obtained *t*-values equal or greater than the true *t*-value represents the likelihood of obtaining the *t*-statistic under the null hypothesis, i.e. the *p*-value. Similarly, the random permutation based ANOVA randomized labels within each participant but otherwise in an unrestricted way across experimental factors [39]. ANOVA *p*-values were Bonferroni corrected for two ROIs and within each ROI *t*-test *p*-values were corrected for three comparisons. Only the corrected *p*-values are reported.

It has recently been argued that doing region of interest analysis with the same ROI across the whole group of participants is a statistically insensitive procedure [40]. Therefore we complemented our previous ROI analysis with a functional ROI (fROI) analysis using the spm_ss toolbox [40]. For each subject separately, we extracted the top 10% of voxels (357 voxels) in the left IFG (pars opercularis and pars triangularis, taken from the AAL template) which exhibited the highest *t*-values in the OR > SR contrast (averaged across music conditions). Strictly speaking the voxels did not need to be adjacent, but in practice they mostly are. In order to ensure the independence of data for fROI identification and activity estimation, we used either the first or the second scanning run for fROI building and the left-out run for estimation of activation during the conditions of interest (see [41]). Responses were averaged across the two partitions (‘ 2-fold cross-validation procedure’, see [42] for a similar approach). Thus, each subject had a different fROI in Broca’s area. Data from the fROI were used to derive average contrast values across voxels. Inferential analyses were again carried out using random permutation based tests and *t*-test *p*-values were corrected for three comparisons (Bonferroni method). Only the corrected *p*-values are reported.

## Results

### Behavioral Results

Participants answered one comprehension prompt after each trial. The accuracy rates revealed that no participants scored at or below chance level, i.e. not with an accuracy below 56% (binomial distribution, *p* < .05). Scores ranged between 66% and 90% (*M* = 78%). A 2 (prompt type: matrix or relative clause) × 2 (language: SR or OR) × 3 (music: in-key, out-of-key, or auditory anomaly) dependent ANOVA revealed three effects. First, there was a main effect of prompt type [*F*_(1,18)_ = 143.56, *p* < .001, _p_η^2^ = .889, _p_ω^2^ = .882], such that prompts targeting main clause understanding were easier to answer (88%) than prompts targeting relative clause understanding (68%). Furthermore, a main effect of linguistic condition was found [*F*_(1,18)_ = 43.90, *p* < .001, _p_η^2^ = .709, _p_ω^2^ = .693] indicating that prompts after SR sentences were answered more accurately (86%) than those after OR sentences (70%). Furthermore, these two main effects interacted [*F*_(1,18)_ = 51.18, *p* < .001, _p_η^2^ = .740, _p_ω^2^ = .725]. Follow-up *t*-tests revealed that the difference between SR and OR sentences is significant for both kinds of prompts albeit larger for those targeting relative clause comprehension [*t*_(18)_ = 7.05, *p* < .001] than those targeting main clause comprehension [*t*_(18)_ = 3.85, *p* < .01]. This supports the idea that OR sentences were indeed more challenging than SR sentences. However, this difficulty did not interact with the music factor [*p* > .3]. The three-way interaction was not significant. It should be borne in mind that the behavioral measure was designed to ensure adequate neural processing instead of showing the previously reported behavioral interaction effect [3]. Therefore, the crucial test of our hypothesis lies in the neural data analysis. We will return to this point in the discussion section.

### fMRI Results

#### Whole-brain Analysis

**Fig 2:**
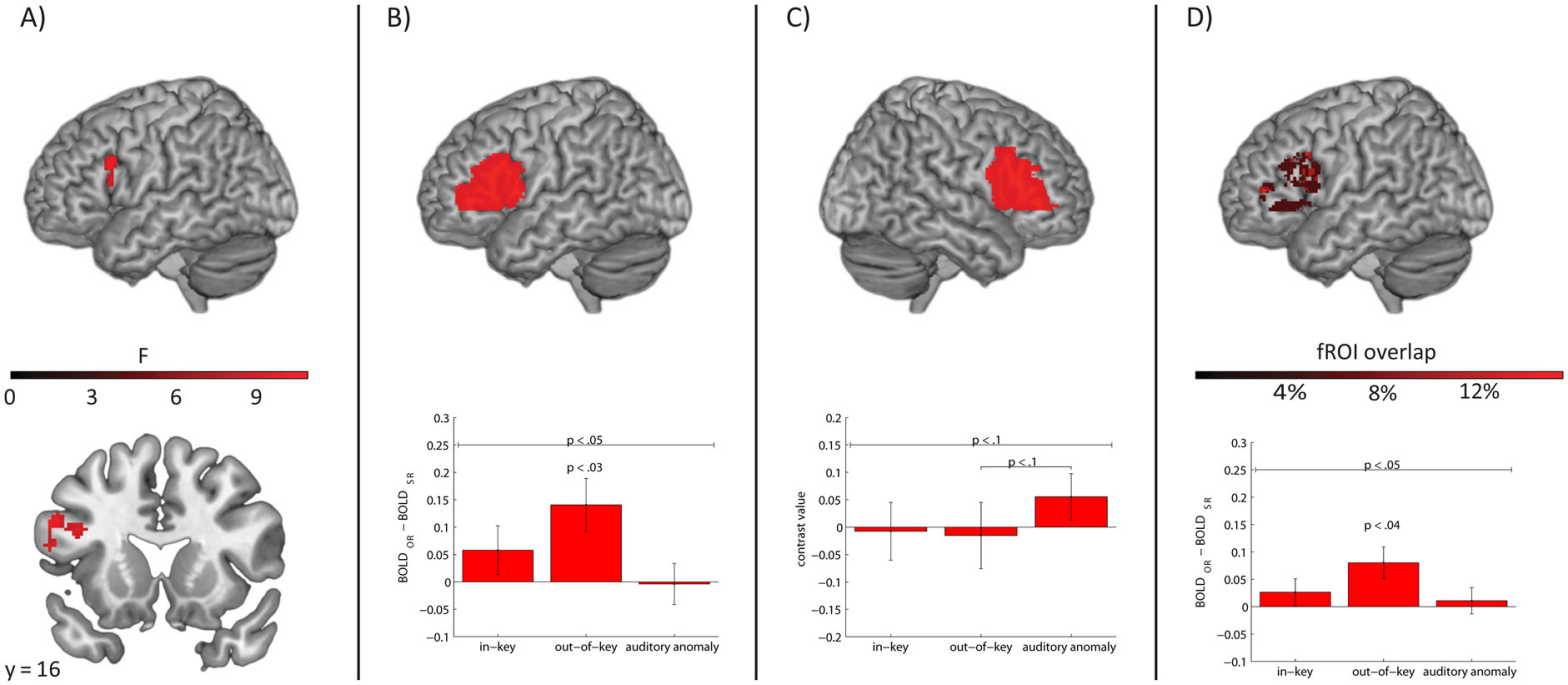
fMRI results. A) The language main effect (OR > SR) found in the whole-brain analysis (p < .005 uncorrected, cluster size = 87 voxels). B) Left hemisphere structural ROI. The BOLD effect of the linguistic manipulation is shown (OR—SR) with the associated *p*-value of a paired *t*-test above the bar. The significance level of the interaction effect is denoted above the line. Bars represent the activity difference (OR-SR) to sequences in which the stressed syllable of the critical word was sung in-key, out-of-key or unusually loudly (auditory anomaly). C) Right hemisphere structural ROI. The BOLD effect (compared to implicit baseline) is shown for each music condition. The *p*-value of a dependent *t*-test comparing two music conditions can be seen above the respective bars. The significance level of the music main effect is denoted above the line. D) Left hemisphere functional ROI. fROIs were individually defined in the left structural ROI. The inter-subject overlap in fROI locations is shown in the top panel. See methods for details. The BOLD effect is shown for the three different music conditions separately. Error = SEM. All *p*-values in structural ROI analyses are Bonferroni adjusted.

For the whole brain analysis, no cluster emerged for any of the main effects or their interaction with a probability of *p* < .05 (FDR corrected). In order to see whether our data set replicates previous findings of language syntax-related effects in left prefrontal areas, we lowered the statistical threshold (*p* < .005 uncorrected) and identified the biggest cluster (87 voxels) with a peak at [-54; 18; 28], see Fig 2A. The cluster represents increased activity to OR sentences compared to SR sentences and it covers parts of the IFG pars opercularis and pars triangularis, showing that our data set can replicate previous findings albeit only at a reduced statistical threshold.

#### Structural ROI analysis

The predicted interaction between the language and music factors was found in left IFG [*F* = 4.14, *p* < .05] but not right IFG [*F* = 1.68, *p* > .4]; see Fig 2B. Follow-up *t*-tests showed that the significant interaction in left Broca’s area emerged because the OR > SR contrast was only significant in the out-of-key condition [*t* = 2.93, *p* < .03] but not in the in-key condition [*t* = 1.30, *p* > .5] or the auditory anomaly condition [*t* < 1]. Similar analyses in the right ROI revealed no significant OR > SR effect in any of the music conditions [all *p* values > .2]. The language main effect was not significant in either region of interest [left: *F* = 4.00, *p* > .1; right: *F* = 1.76, *p* > .4]. However, the music main effect was marginally significant in the right hemisphere region of interest [*F* = 3.26, *p* < .1] but not in the left one [*F* = 2.63, *p* > .1]; see Fig 2C. The former was due to a marginally greater activation in the auditory anomaly condition compared to the out-of-key condition [*t* = 2.28; *p* < .1]. The contrast with the in-key condition did not approach significance [*t* = 2.00; *p* > .1], nor did the in-key vs. out-of-key contrast [*t* < 1].

#### Functional ROI analysis

The 10% of voxels in the left IFG which exhibited the strongest language effect were used to construct an fROI for each subject separately. Fig 2D (top panel) shows that the overlap of included voxels was small across participants, reflecting known individual differences in the location of language-related activity peaks in Broca’s area [43]. Similar to the structural ROI, this language syntax-related fROI exhibited the predicted interaction between the language and the music factors [*F* = 3.27, *p* < .05], see Fig 2D (bottom panel). Follow-up *t*-tests again showed that the significant interaction in the fROI emerged because the OR > SR contrast was only significant in the out-of-key condition [*t* = 2.78, *p* < .04] but not in the in-key condition [*t* = 1.09, *p* > .5] or the auditory anomaly condition [*t* < 1]. The language main effect—indicative of an OR > SR pattern [*F* = 3.89, *p* < .1]—as well as the music main effect [*F* = 2.65, *p* < .1] were only marginally significant. The latter was reflecting a pattern previously seen in the right hemispheric structural ROI: greater activity to the auditory anomaly condition compared to the other two music conditions.

## Discussion

The present study aimed to provide brain-imaging support for the proposal that syntax processing in music and language interact in the human brain. To this end we adopted an interference paradigm. We found a statistical interaction between music and language processing in Broca’s area, corresponding to BA44 and BA45 in the left inferior frontal gyrus. This music-language interaction even emerged when restricting the analysis to voxels within Broca’s area which are involved in language syntax processing. This suggests that at least some of the neural resources in Broca’s area that process syntactic relations between words in language are also sensitive to syntactic relations between tones in music, and that syntactic integration in language is not wholly independent of syntactic integration in music. Note that this non-independence is not due to shared general attention resources as an auditory anomaly led to a different activation pattern.

Specifically, the interaction between music and language emerged when participants heard a stimulus containing a syntactically challenging sentence (object-extracted relative clause instead of subject-extracted relative clause) sung on a melody containing a syntactically challenging tone (out-of-key instead of in-key), with the tone located at the precise point in the melody where the linguistic syntactic integration difficulty occurred. In this case an interaction pattern emerged (see Fig 2B and 2D). This is indicative of an even greater integration difficulty in this condition compared to what would be expected from integrating challenging words and tones entirely independently of each other. In order to check whether non-syntactic auditory anomalies would also show such a pattern we included a control condition in which the critical tone was sung in-key but unusually loudly. In Broca’s area this control condition did not lead to activity patterns similar to the out-of-key condition, even in numerical terms. Instead of affecting the left hemisphere Broca’s area, the control condition seemed to activate the right hemisphere homologue of Broca’s area. The implications of these findings for our understanding of music and language are discussed below.

### A Common Role for Broca’s Area in the Music and Language Networks

The current study has found some support for a common syntactic processing role of Broca’s area in music and language. This fits with results showing that musical training is associated with structural changes in this area [44–47] and altered language syntax processing [48,49]. Damage to this brain area is also known to lead to processing deficits in both language and music in non-musicians [26].

However, the results of four recent fMRI studies might appear to contradict a common role for Broca’s area in the music and language networks. Two found common brain areas but differing music and language activation patterns in them using multi-voxel pattern analysis (MVPA) [29,50]. In two other studies Fedorenko et al. [42,51] found different activated brain regions when comparing a music-localizer based on a scrambling manipulation to a language-localizer based on the reading of sentences versus lists of non-words. However, none of these studies specifically manipulated syntactic structure in language and tonal/harmonic structure in music, while leaving other aspects of sequence structure intact (cf. [52,53]). It is also worth keeping in mind that the SSIRH actually predicts the overlap between music and language to be partial, not complete. The question which we attempted to answer in this study was whether music and language share *any* circuitry at the level of syntactic processing, as suggested by the music-language interaction in Broca’s area.

### Non-syntactic Overlap between Music and Language

Despite the evidence for a shared syntactic processing mechanism in the left inferior frontal gyrus, alternative explanations for our results could be proposed. First of all, any auditory anomaly might draw attention away from language and thus interact with linguistic processing. We reject this explanation because a control condition consisting of a sudden 10dB loudness increase did not lead to a similar pattern of results compared to the harmonic violation. This is striking since, as opposed to the subtle harmonic violation which interacted with language processing, the loudness increase evoked a marginally significant brain correlate in the right hemisphere’s inferior frontal gyrus. This more salient non-syntactic manipulation, however, did not interact with language processing. This supports a shared syntactic neural architecture between music and language. Furthermore, the finding is in line with previous behavioral and ERP studies which found that neither a loudness anomaly nor a timbral anomaly leads to the same music-language interactions as seen with harmonic manipulations [3,16,18].

Another recent alternative explanation has been Rogalsky et al.’s [29] proposal that music and language processing exhibit a link only in tasks which involve the processing of violations. However, the current study elicited a music-language interaction by using relatively easier or more difficult linguistic constructions which were without any errors, as well as a musically plausible in-key/out-of-key tone manipulation [33]. Moreover, the brain activation response we find is not indicative of linguistic error processing which is associated with relatively more right-lateralized prefrontal activation sites [54], as opposed to the relatively left-lateralized effect here. Thus, the overlap we found does not appear to be elicited only under the exceptional circumstances of processing violations (see also behavioural studies without error manipulations [3,18]).

Still, some studies have reported interactions between music and semantic language manipulations [17,55]. The present study does not directly address semantic language processing, but its design could be extended to investigate the neural differences between semantic-harmonic and syntactic-harmonic interactions. Thus, more research is required in order to address the syntax-specificity of the interaction we found in the present study.

Besides attention and violation processing, it could also be suggested that the observed activation differences reflect decision-making related processes. Binder et al. [56] have shown that a cluster in the lateral part of the left IFG is associated with decision making performance in a syllable differentiation task. However, such an explanation is unlikely to reflect the pattern seen here because a decision was only required *after* a song was heard, upon seeing a comprehension prompt. Furthermore, the kind of prompt was variable and unpredictable. For example, half the comprehension prompts did not focus on the relative clause manipulation at all. Thus, activation differences due to a decision process are unlikely as decision making started after the song, i.e. after the time interval which the current fMRI analysis investigated.

### The Role of the Right Inferior Frontal Gyrus

The right hemisphere homologue of Broca’s area did not show activity related to linguistic or harmonic processing, which was partly surprising given that previous brain imaging studies reported an involvement in both cases (e.g., [27,28]). In contrast to these studies, we employed a task-unrelated, subtle harmonic manipulation which was based on a single tone (the smallest possible alteration of melody). This manipulation might not have been strong enough to reliably activate right hemisphere areas involved in musical harmonic processing (e.g., [28]). In future work, one could increase the salience of the tonal/harmonic manipulation, e.g., by using a melody sung over instrumental musical chords, or over an instrumental melody with several notes per sung word, so that the critical word was accompanied by several out-of-key notes. The subtle effect we find could be taken to suggest that the tone manipulation is only able to modulate linguistic processing already triggered by the language task. Instead of syntactic or harmonic processing we found a marginal attention-related effect in the right inferior frontal gyrus. Our control condition, a salient loudness increase, seemed to activate this region, likely due to its involvement in the bottom-up attention network [57,58].

### Limitations

The absent behavioural effect of music on language might appear surprising. However, it does not necessarily contradict the proposal for shared syntactic processing resources in the left inferior frontal gyrus. In comparison to a previous behavioral study [3] which did find a behavioral effect with a similar paradigm, several aspects of our study might have lowered the sensitivity of our behavioral measure. First, we used a diverse set of comprehension prompts which were partly challenging in themselves (e.g., passive voice prompts) and which often did not focus on the relative clause manipulation. This was necessary in order to discourage unnatural task strategies, a problem Fedorenko et al. [3] were not faced with due to linguistic differences such as a word order manipulation in English vs. a number agreement manipulation in Dutch. Second, in order to reduce the duration of scanning sessions, our stimulus list did not include fillers. Third, our stimuli were rhythmically and linguistically more diverse, possibly increasing ecological validity at the expense of reducing the effect size. In sum, by focusing the present study on exploring a neuronal effect we did not optimize the design for finding a behavioral effect.

The neural effects we find could appear weak. Concerning the language main effect, we only find a marginally significant language syntax effect in Broca’s area, and that result only emerges in the functional region of interest analysis. This weak effect might be a consequence of the syntactic manipulation we used. Dutch participants could have misheard the number of the relative clause verb in the more difficult object-extracted relative clauses, and therefore ‘default’ to the more common subject-extracted relative clause version. Such a process is considerably less likely in an object- versus subject-relative clause manipulation based on word order, such as used in [3]. Thus, future work might employ a word-order based syntactic contrast. Similarly, the influence of music on the language effect was relatively weak, see Fig 2B and 2D. This might simply mirror the rather subtle music manipulation in combination with our particular choice of syntactic constructions. Moreover, pilot work reported by Fedorenko et al. [3] suggests that music-language interaction effects might be enhanced by an increased rate of presentation (the average rate in [3] was 1.78 words/sec, versus 0.98 in the current study). Future work is needed to test whether the effects found here generalize to other music and language manipulations.

## Conclusion

The present study aimed to test the hypothesis that music and language share neural resources for syntactic processing in Broca’s area. The predicted interactive pattern between music and language demands was indeed found in this part of the brain. This is the first direct evidence which suggests that music and language syntactic processing interact in Broca’s area.
